# Efficiency and Power Limits of Electrical and Tendon-Sheath Transmissions for Surgical Robotics

**DOI:** 10.3389/frobt.2018.00050

**Published:** 2018-06-18

**Authors:** Christopher R. Wagner, Evangelos Emmanouil

**Affiliations:** Medical Technology, Cambridge Consultants, Cambridge, United Kingdom

**Keywords:** surgical robotics, tendon sheath transmissions, cable drives, efficiency, minimally invasive surgery, shape memory alloy (SMA)

## Abstract

A popular design choice in current surgical robotics is to use mechanical cables to transmit mechanical energy from actuators located outside of the body, through a minimally invasive port, to instruments on the inside of the body. These cables enable high performance surgical manipulations including high bandwidth control, precision position control, and high force ability. However, cable drives become less efficient for longer distances, for paths that involve continuous curves, and for transmissions involving multiple degrees of freedom. In this paper, we consider the design tradeoffs for two methods of transmitting power through an access port with limited cross sectional area and curved paths - tendon/sheath mechanical transmissions and electrical wire transmissions. We develop a series of analytic models examining fundamental limits of efficiency, force and power as constrained by access geometry, material properties, and safety limits of heat and electrical hazards for these two transmission types. These models are used to investigate the potential of achieving the required mechanical power requirements needed for surgery with smaller access ports and more difficult access pathways. We show that an electrical transmission is a viable way of delivering more than sufficient power needed for surgery, highlighting the opportunity for next-generation actuators to enable more minimally invasive surgical devices.

## Introduction

Current generation laparoscopic surgical robots, such as the daVinci Xi (Intuitive Surgical, Sunnyvale, CA, USA) are high performance general purpose machines for supporting surgeons in executing surgical tasks. They allow for high bandwidth control, precision position control, high force ability, and high endurance (Taylor et al., 2016). To achieve these benefits, surgical robots use rigid shafts in combination with mechanical cables around pulleys to transmit mechanical energy from outside of the body to the distal joints inside the body. This is an effective and efficient transmission for straight-line access from the entry port to the surgical site when using stiff materials and low friction bearings.

**Figure 1:**
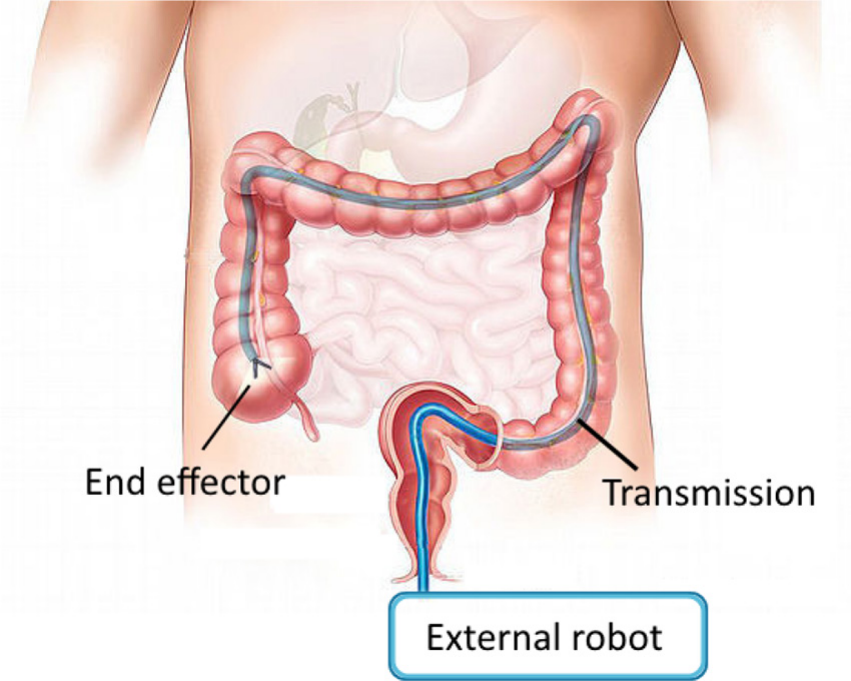
Schematic of a curved path MIS robotic system. Manipulations carried out at the end effector are powered through a transmission which passes through access geometry which is restricted.

Access is limited, however, to a straight line from entry point to relevant anatomy. We would like to extend the minimally invasive benefits of surgical robotics - increasing dexterity with reduced trauma and reduced healing time - to more surgical procedures that involve longer and more tortuous access pathways (Figure 1). Candidates include natural orifice transluminal endoscopic surgery (NOTES) and augmenting manipulation capabilities for flexible endoscopic procedures (McGee et al., 2006; Burgner-Kahrs et al., 2015). Additional procedures, such as vascular access, or additional benefits, such as sutureless entry wounds, can also be considered if the access port diameter were further reduced from its current minimum of 5 mm (Ferguson and O’Kane, 2004; Tacchino et al., 2009).

There are several approaches to deliver the mechanical energy needed to carry out surgical manipulations from the outside of the body to the inside. One approach is to use a tendon/sheath drive - continue to use mechanical cables, but embedded in a stiff sheath that can provide the reaction force against which to actuate the tendons. This approach has the obvious limitation that as the access diameter is reduced, and the access path length increases and becomes curvier, the performance of the transmission will decrease due to friction, stiffness and inertia of the tendon. Further, there becomes a tradeoff with number of achievable degrees of freedom as each requires additional tendons which require additional cross sectional area.

An alternate transmission that would not suffer from these access path limitations is an electrical transmission - a wire. The efficiency of a wire to transmit power is high; however, creating a minimally invasive surgical robot based on an electrical transmission has the obvious drawback that the actuators now need to be located on the inside of the body. Examples of this approach exist (Takayama et al., 1997; Mineta et al., 2001; Yeung and Gourlay, 2012; Lee et al., 2014), though the actuator size is now the dominant factor. Still, if the actuators of sufficient performance could be placed significantly closer to the surgical site inside the body, this would remove another limitation of current surgical robots - the large size of the systems. Actuators on the inside of the body would likely not suffer from gravitational effects nearly as much; the knock-on effect of a small increase in distal actuator size resulting in a larger set of proximal actuators for a serial arm configuration would be avoided. And, there are plausible actuator technologies that have a significantly higher work density that traditional electrical motors, including shape memory alloy and piezoelectrics (Huber et al., 1997), that would achieve sufficiently low internal actuator volumes.

In this paper, we present a series of analytic models that investigate the design tradeoffs involved in considering these two transmission approaches for surgical robotics. We first establish a model describing the limits of heat uptake in the body, which is the fundamental limit for both transmission types. We then develop a model of tendon/sheath power transmission, establishing limits of efficiency, force, and power based on access geometry and material properties. Similarly, we develop a model for electrical transmission of power into the body, taking into account access geometry and electrical safety limits to identify efficiency and power limits. Using these models, we then evaluate their relative ability to efficiently deliver power and required performance under the access constraints posed by minimally invasive surgery. The results highlight the overall opportunity for high performance minimally invasive robotics with more stringent access geometries than current systems.

## Heat Dissipation as Fundamental Limit to Power Delivery

A fundamental limiting factor when delivering power into the human body is the corresponding power lost to heat along the length of the transmission. If the temperature rises too high, cell death and permanent tissue damage can result (Rossmanna and Haemmerich, 2014). Medical device regulations provide guidance as to safe temperature limits that can be applied to the body (International Electrotechnical Commission, 2014), depending on the length of applied time. However, these temperature limits need to be translated into power limits, as parameterised by tissue thermal properties, to be useful as design guidance.

In this section, we present an analytic model of local heat propagation to relate known safe temperature limits to corresponding power limits. These power limits are used in the transmission models presented in later sections. The following model derives thermal power limits for a cylindrical geometry (such as a mechanical or electrical wire) giving off heat into surrounding tissue in steady state, based on a solution to the well known bioheat equation (Incropera et al., 2011). While other bioheat models exist, the use of the bioheat equation is widespread and a range of measurements exist for the model constants for different tissues (Kerdok et al., 2006; Hasgall et al., 2015). Also, use of the bioheat equation encapsulates the key properties of heat removal in bulk tissue (diffusion and heat loss through capillary perfusion) and does not require us to make an estimate of an imprecise convection term.

This model is useful as an initial estimate for heat limits, and its analytic nature is useful on which to base further calculation. For more complicated geometries and tissue interactions, a mesh style solver may be required to derive more precise limits.

As derived more fully in the Appendix, an estimate for the upper bound of heat power that the body can safely dissipate through a cylinder of radius r, per unit length, is given by

(1) $$H_{max}(r)=2\pi kBr\frac{K_{1}(Br)}{K_{0}(Br)}(T_{1}-T_{a})$$

where

(2) $$B=\sqrt{\frac{\omega \rho_{b}c_{b}}{k}}$$

and $\omega ,\rho_{b},c_{b},k$ are tissue thermal properties as described in Table 1, $T_{1}$ is the surface temperature of the cylinder, and $K_{0}(...)$ and $K_{1}(...)$ are zeroth and first order modified Bessel functions of the second kind, respectively.

**Table 1:** Thermal Nomenclature.

Symbol	Definition	Unit
r	Outer radius of tool or wire	m
w	Width of insulation	m
l	Length of wire	m
$T_{a}$	Arterial or body temperature	${}^{\circ }C$
$\rho_{b}$	Density of blood	$kg/m^{3}$
ω	Perfusion rate	$m^{3}/(s\cdot m^{3})$
$c_{b}$	Specific heat of blood	J/(kg⋅K)
k	Thermal conductivity	W/(m⋅K)
$I_{n}(...)$	*n*th order Modified Bessel Function of the first kind	N/A
$K_{n}(...)$	*n*th order Modified Bessel Function of the second kind	N/A
$\rho_{wire}$	Resistivity of wire	Ω⋅m
$T_{1}$	Surface temperature of the cylinder	${}^{\circ }C$
$H_{max}(r)$	Maximum heat power the body can safely dissipate	W
${\dot{q}}_{m}$	Metabolic heat	$W/(m^{2}s)$

## Efficiency Limits of Mechanical Tendon/sheath Transmissions

Current laparoscopic surgical robots use mechanical cable drives for tool actuation inside the body as well actuating external body motion. These systems rely on tense cables running between pulleys, which are well modelled in the literature (Miyasaka et al., 2015; Miyasaka et al., 2016) to enable higher performance through closed loop control (Haghighipanah et al., 2015; Rosen et al., 2017). Fundamental efficiency limits of this style of cable/pulley transmission have also been established (Townsend and Salisbury, 1988), as well as size and parameter tradeoffs as applied to surgical tools (Friedman, 2011).

However, the principles of operation of cable/pulley transmissions do not directly extend to curved paths. Instead, use of a mechanical cable drive through a continuous curved path requires use of a sheath around the cable to provide opposing tangential and axial forces. Models for friction and control of these systems exist in the literature, with varying levels of detail relating to complex effects such as tendon viscoelasticity and hysteresis (Palli and Melchiorri, 2006; Palli et al., 2009; Agrawal et al., 2010; Palli et al., 2012; Do et al., 2015; Choi et al., 2017). We extend here a simple tendon/sheath model (Palli and Melchiorri, 2006; Palli et al., 2009), in combination with conservative loss assumptions, to derive power and efficiency limits. Based on these limits, we can compare the performance of cable/sheath transmissions to other transmissions, especially in cases where a cable/sheath approach may intuitively seem less efficient, such as for long, curvy paths. Key to these models are the incorporation of the design constraints that will relate to MIS surgery, such as access diameter and path length, as well as performance outputs, such as efficiency, force, and degrees of freedom.

### Efficiency Model

**Figure 2:**
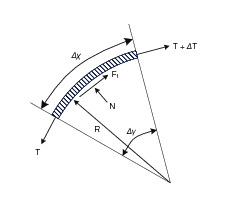
Tendon segment force balance model.

In this section, we derive an expression for the efficiency limits of tendon/sheath transmissions, based on validated models that exist in the literature. We start with a simple tendon/sheath model that relates input and output tensions as parameterised by a radial path geometry (Figure 2, Table 2), and a friction coefficient relating tendon tension with friction forces (Palli and Melchiorri, 2006; Palli et al., 2009). A single parameter (curvature) is used to parameterise the radial path geometry, where more complex paths can be modelled with a simple radial path with equivalent accumulated angle (Do et al., 2015). This model also accounts for tendon stretch but does not assume any stretch or losses due to the sheath. Because we are only concerned with power and efficiency limits and not more complex effects such as tendon hysteresis, we make a conservative assumption where we only transmit energy when pulling and all energy associated with hysteresis is assumed lost. We re-derive the solution to the model to be explicit about the contribution of pretension so that it can be correctly incorporated into an expression for efficiency.

**Table 2:** Mechanical Nomenclature.

Symbol	Definition	Unit
$P_{target}$	Upper bound mechanical power target	W
F	Force	N
v	Velocity	m/s
ΔT	Change in tension	N
$F_{f}$	Force due to friction	N
μ	Tendon sheath friction coefficient	N/A
N	Normal force	N
$\dot{\epsilon }$	Tendon velocity	m/s
Δγ	Tendon subtended angle	rad
Δx	Tendon section length	m
R	Radius of curvature of the tendon section	m
$T_{x}$	Tendon tension at x	N
$T_{in}$	Input tension at x=0	N
$T_{out}$	Output tension at x=L	N
δ	Tendon elongation	m
E	Modulus of elasticity of the tendon material	N/A
A	Cross sectional area of tendon or wire	$m^{2}$
L	Tendon sheath length	m
$T_{in,w}$	Input tension associated with work	N
$T_{out,w}$	Output tension associated with work	N
$T_{0}$	Preload tension	N
$\delta_{0}$	Tendon elongation due to preloading	m
$\delta_{w}$	Tendon elongation due to work tension	m
D	Input motion distance	m
$W_{in}$	Work in	J
$W_{out}$	Work out	J
η	Power transmission efficiency	N/A
$\eta_{lim}$	Power transmission efficiency as D→inf	N/A

As derived more fully in the Appendix, we separate an explicit pretension term ($T_{0}$) from the input and output tendon tensions:

(3) $$\begin{array}{c} T_{in}=T_{in,w}+T_{0}\\ T_{out}=T_{out,w}+T_{0} \end{array}$$

The output tension capable of doing work $T_{out,w}$ is given by

(4) $$T_{out,w}=T_{in,w}e^{-\frac{\mu L}{R}}-T_{0}\left(1-e^{-\frac{\mu L}{R}}\right)$$

and the stretch of the tendon $\delta_{w}$ only due to input tension (and not pretension) is

(5) $$\delta_{w}=\frac{T_{in,w}R}{EA\mu }\left(1-e^{-\frac{\mu L}{R}}\right)$$

We can now write the relationship for efficiency, relating work in to work out. We do not need to include the prestretch, and use $T_{in,w}$ and $T_{out,w}$ as the input and output tensions that account for transmitted work. Assuming an input motion of distance D at tension $T_{in,w}$, then

(6) $$W_{in}=DT_{in,w}$$

and

(7) $$W_{out}=(D-\delta_{w})T_{out,w}$$

so efficiency η becomes

(8) $$\begin{array}{ll} \eta \;\;\;\; & =\frac{W_{out}}{W_{in}}\\ & =\frac{\left(D-\delta_{w}\right)T_{out,w}}{DT_{in,w}} \end{array}$$

Expanding terms and simplifying gives a final equation for efficiency:

(9) $$\begin{array}{ll} \eta = & e^{-\frac{\mu L}{R}}(1-\frac{T_{0}}{T_{in,w}}(e^{\frac{\mu L}{R}}-1)\\ & -\frac{R}{EA\mu D}(T_{0}(2-e^{\frac{\mu L}{R}}-e^{-\frac{\mu L}{R}})+T_{in,w}(1-e^{-\frac{\mu L}{R}}))) \end{array}$$

Examining (9), we note that as D approaches infinity, the efficiency limit can be simplified to:

(10) $$\eta_{lim}=e^{-\frac{\mu L}{R}}(1-\frac{T_{0}}{T_{in,w}}(e^{\frac{\mu L}{R}}-1))$$

which corresponds to the overall work delivered being large compared to tendon losses. This applies to a tendon/sheath drive where power is transmitted continuously in one direction, such as in a closed loop. Note that this expression is not simply the tension ratio of $T_{out,w}$ to $T_{in,w}$, but contains an explicit term that decreases efficiency with the increase of pretension.

### Degrees of Freedom and Bend Radius

This analysis is meant to support an understanding of the achievable performance for a given cross sectional access area, path geometry, and material property limits. As observed above, the efficiency of a tendon drive relates to the cross sectional tendon area, where larger tendons of the same material result in stiffer and thus more efficient tendon drives. To relate this result to the total cross sectional access area, we also need to account for sheath stiffness as well as degrees of freedom.

We make the simplifying assumption that sufficient sheath material must exist so that the sheath stiffness must at least match the tendon stiffness (when loaded axially). If this was not the case, the sheath stiffness would dominate and performance would be limited.

Using this assumption, and the limit that $n_{dof}$ degrees of freedom can be controlled by as few as n+1 tendons (Tsai, 1999), this gives an upper bound to the number of degrees of freedom achievable for a given access area. Solving the following set of equations:

(11) $$\begin{array}{rl} E_{sheath}A_{sheath} & =n_{tendon}E_{tendon}A_{tendon},\\ A_{total} & =A_{sheath}+n_{tendon}A_{tendon},\\ n_{dof} & =n_{tendon}-1 \end{array}$$

and using a cylindrical expression for area gives an upper bound estimate for the number of achievable degrees of freedom for a tendon sheath system, for a given geometry and material properties.

(12) $$n_{dof}=\frac{r^{2}E_{sheath}}{r_{tendon}^{2}\left(E_{tendon}+E_{sheath}\right)}-1$$

This expression is a conservative bound, as it does not account for additional area needed for sliding tolerances, working channels, or close packing adjustments. Also, an equal stiffness assumption was used to relate the cross sectional area devoted to the sheath material versus tendon material; more complex failure modes like buckling are not accounted for. Further, if this limit is used, it assumes a monolithic sheath whose bending is limited by the material properties, not by additional geometry features (such as notches to increase flexibility). Finally, a common design simplifying control of the system uses 2 tendons for each degree of freedom, which further exacerbates the conservative bound.

If we restrict ourselves to material strain limits, then an estimate of the corresponding minimal bend radius relative to tool radius can be calculated using estimates of strain at yield:

(13) $$R_{min}=\frac{r}{\epsilon_{yield}}$$

### Power and Force Limits

Power delivery through a tendon sheath system is limited by the maximum force achievable per tendon, and the maximum velocity achievable per tendon without causing heat damage due to frictional losses.

Force limits for wire rope can be modelled with an exponential fit relating radius to breaking strength; see (Friedman, 2011) for experimental fits to several material types. The working limit of a wire rope is then related to the breaking limit through a safety factor. An expression for this working limit tension $T_{WL}$ is given by

(14) $$T_{WL}=\frac{1}{s}\alpha r_{tendon}^{\beta }$$

where α and β are the breaking limit fit parameters, $r_{tendon}$ is the radius of the tendon, and s is the safety factor.

The maximum velocity for a tendon (at this maximum tendon force, delivering maximum power) can be derived from the cable and heat models given above. We observe that the power loss between input and output should never exceed the heat limit of the tissue through the sheath. For large motions, this can be expressed as

(15) $$T_{in}v-T_{out}v\leq H_{max}(r)L$$

where v is the velocity of the tendon, and L is the path length. Note that we use $T_{in}$ and $T_{out}$ which incorporates pretension, instead of $T_{in,w}$ and $T_{out,w}$, as the heat loss depends on the total tension, not just the delivered power.

Because $T_{o}ut$ exponentially decreases along the length of the path, the point that will have the most loss due to friction will be at the beginning, where absolute tension and loss per unit length is greatest. Thus, we can derive the maximum allowable tendon velocity by taking the derivative with respect to path length of the above expression, and solving for v at L = 0.

(16) $$\begin{array}{rl} \frac{d\left(T_{in}v-T_{out}v\right)}{dL} & =\frac{d\left(H_{max}\left(r\right)L\right)}{dL}\\ -\frac{dT_{out}}{dL}v & =H_{max}\left(r\right)\\ T_{in}e^{-\frac{\mu L}{R}}\frac{\mu }{R}v & =H_{max}\left(r\right) \end{array}$$

Evaluating at L = 0, using $T_{WL}$ as the maximum input tension, and solving for v gives an expression for the maximum allowable velocity $v_{max}$:

(17) $$v_{max}=\frac{H_{max}(r)R}{\mu T_{WL}}$$

For this expression note that r is the outer radius of the sheath (or sheath bundle), not the radius of the tendon material.

## Efficiency Limits of Electrical Cables

In this section, we develop a similar analytic model to investigate the ability of an electrical wire to transmit power within the body, subject to an access geometry constraint. The model also takes into account basic electrical safety constraints which would apply when transmitting power into the body; namely, electrical breakdown causing current flow through tissue.

### Coaxial Wire Model

**Figure 3:**
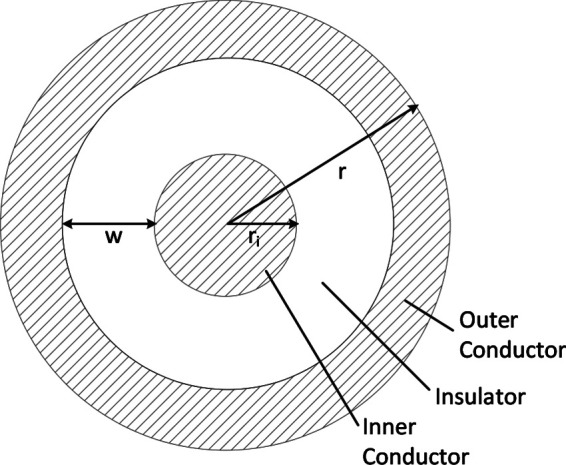
Coaxial wire model.

For this analysis, we use a coaxial wire structure (Figure 3) to account for the voltage and return carrying lines, as this provides the opportunity for increased safety for power delivery in the body if the outer conductor is at the same voltage as the body. A full safety analysis, however, including additional mitigations such as galvanic isolation and over-current detection is outside the scope of this work.

In this coaxial model, the outer radius is r, the radius of the inner conductor is $r_{i}$, and the cross sectional area of the two conductors are equal to account for the return path of the current (Table 3). Thus, the width of insulation w can be calculated from equating the conductor areas

**Table 3:** Electrical Nomenclature.

Symbol	Definition	Unit
$r_{i}$	Radius of inner conductor	m
w	Insulation width	m
$\rho_{wire}$	Resistivity of wire material	Ωm
$R(r_{i},l)$	Wire resistance	Ω
$P_{heat}(r,r_{i},l)$	Thermal power dissipated by wire	W
$I(r,r_{i},l)$	Electrical current through wire	A
$V_{breakdown}$	Breakdown voltage	V
d	Dielectric constant of insulator material	F/m
$V_{operating}$	Operating voltage	V
s	Safety factor	N/A
$P_{max}$	Maximum input power	W
$P_{max,out}$	Maximum output power	W
$\eta_{wire}(r)$	Power transmission efficiency of wire	N/A
Λ	Constants expression	N/A

(18) $$\pi r^{2}-\pi {\left(r_{i}+w\right)}^{2}=\pi r_{i}^{2}$$

and solving for w. This gives

(19) $$w=\sqrt{r^{2}-r_{i}^{2}}-r_{i}$$

Also note that for an actual wire used in the body, an additional biocompatibility layer would need to exist outside of the outer conductor. This layer has been omitted in this model because it can be made thin (e.g., less than 10 um) in practice.

### Current Limits

Given the above limit of heat into the body along a cylinder, we can derive the maximum current through the wire by Joule heating using the resistivity of the wire. Given that the absolute resistance of a wire is related to the length of the wire, we start with all equations taking into account the length explicitly.

So, from above:

(20) $$H_{max}(r,l)=lH_{max}(r)$$

Resistance of a wire R is proportional to length and the resistivity of the material ($\rho_{wire}$), and inversely proportional to area. In the coaxial wire model, the cross sectional area of the current conducting portions of the wire is given by $A=2\pi r_{i}^{2}$. The resistance equation then becomes

(21) $$R(r_{i},l)=l\frac{\rho_{wire}}{A}$$

(22) $$=l\frac{\rho_{wire}}{2\pi r_{i}^{2}}$$

Assuming the same amount of forward and return current in the separate conductors in the coax wire, the resistivity and heat can be related using an equation for Joule heating:

(23) $$P_{heat}(r,r_{i},l)=2I{\left(r,r_{i},l\right)}^{2}R(r_{i},l)$$

Solving for I, and maintaining our dependence on coax geometry parameters r, $r_{i}$ and l:

(24) $$I_{max}(r,r_{i},l)=\sqrt{\frac{H_{max}(r,l)}{2R(r_{i},l)}}$$

(25) $$=\sqrt{\frac{lH_{max}\left(r\right)}{l\frac{\rho_{wire}}{\pi r_{i}^{2}}}}$$

(26) $$=r_{i}\sqrt{\frac{\pi }{\rho_{wire}}H_{max}(r)}$$

Thus, we observe that the $I_{max}$ does not depend on the length of the wire.

### Voltage Limits

The above analysis determines the maximum heat in the body as limited by the heat dissipation ability of tissue. However, another effect corresponds to limit the voltage used inside the body, which thus limits the maximum power delivered. The risk is that with high voltages, the voltage exceeds the breakdown voltage (dielectric strength) of the insulator, leading to an electrical hazard. Thus, voltages are typically limited inside the body. In the case of the coaxial cable, one form of protection is setting the outer conductor at the same potential as the body. However, we still need to protect against an internal breakdown of the insulator to prevent the outer conductor achieving a high voltage.

The breakdown voltage is related to the material properties of the insulator and the geometry with the following relationship:

(27) $$V_{breakdown}=dw$$

where $V_{breakdown}$ is the breakdown voltage, d is the dielectric constant of the material, and w is the width of the insulation. The maximum operating voltage is usually related to the breakdown voltage with a safety factor (at least a factor of 5 smaller than the breakdown voltage):

(28) $$V_{operating}=\frac{V_{breakdown}}{s}$$

where s is the desired safety factor.

Expressed in terms of our coaxial model geometry parameters, $V_{operating}$ is

(29) $$V_{operating}=\frac{d}{s}\left(\sqrt{r^{2}-r_{i}^{2}}-r_{i}\right)$$

### Power and Efficiency Limits

With the above relationships for voltage and current as related to geometry, we can now derive an estimate for efficiency and power limits. First, we identify the optimal inner conductor radius and corresponding insulator thickness for a given outer radius. As insulator thickness increases, the allowable drive voltage and thus power increases; however, available cross sectional area decreases, causing resistance to increase, which decreases power delivery.

Power can be expressed as the product of $I_{max}$ (which is limited by thermal limits into the surrounding tissue) and $V_{operating}$ (which is limited by breakdown voltage of the insulator and a safety factor).

(30) $$P_{max}(r,r_{i})=I_{max}(r,r_{i})V_{operating}(r,r_{i})$$

We observe that there is an optimal inner conductor radius that exists for each external radius. We can explicitly solve for this $r_{i,max}$ by taking the derivative of the power expression with respect to $r_{i}$, and setting equal to zero.

(31) $$\begin{array}{ll} P_{max} & =\sqrt{\pi }r_{i}\sqrt{\frac{1}{\rho_{wire}}H_{max}\left(r\right)}\frac{d}{s}\left(\sqrt{r^{2}-r_{i}^{2}}-r_{i}\right)\\ \frac{dP_{max}}{dr_{i}} & =\frac{\sqrt{\pi }d\sqrt{\frac{1}{\rho_{wire}}H_{max}\left(r\right)}}{s\sqrt{r^{2}-r_{i}^{2}}}\left(r^{2}-2r_{i}^{2}-2r_{i}\sqrt{r^{2}-r_{i}^{2}}\right) \end{array}$$

We see that $dP_{max}/dr_{i}$ will be zero when

(32) $$\left(r^{2}-2r_{i}^{2}-2r_{i}\sqrt{r^{2}-r_{i}^{2}}\right)=0$$

Solving for $r_{i}$, and choosing the expression that will result in real values yields

(33) $$\begin{array}{c} r_{i,max}=r\sqrt{\frac{1}{2}\left(1-\frac{\sqrt{2}}{2}\right)}\\ \approx 0.38r \end{array}$$

Substituting this result back into our power expression, collecting known constants into a single term Λ and simplifying, we can derive the final expression for $P_{max}$:

(34) $$P_{max}=\frac{\Lambda dr^{2}}{s}\sqrt{\frac{1}{\rho_{wire}}H_{max}(r)}$$

where

(35) Λ≈0.37

Note that $P_{max}$ is the *input* power maximum, which is derived from voltage and heat safety limits which will apply at the beginning of the wire. The power transmitted needs to take into account the losses in the wire, which relates to the length. Since we have derived the limits based on power lost to heat, the output power maximum simply becomes

(36) $$P_{max,out}=\frac{\Lambda dr^{2}}{s}\sqrt{\frac{1}{\rho_{wire}}H_{max}(r)}-lH_{max}(r)$$

Finally, we can write an equation for the efficiency of transmission η, which relates the input power and the power lost to Joule heating in the wire. Note that this relationship does depend on length, as the total power lost to heat increase per unit length of wire.

(37) $$\eta_{wire}(r)=1-\frac{lH_{max}(r)}{P_{max}(r)}$$

## Results

In this section, we evaluate and compare the previously derived models of heat limits, mechanical transmissions, and electrical transmissions using representative values of tissue constants and design parameters that apply to minimally invasive surgery. We first establish the mechanical performance target (including force and power) that a transmission for MIS surgery is attempting to achieve, then explore the ability of mechanical and electrical transmissions to meet that performance target. For both transmissions, we examine the corresponding efficiency for different access constraint geometries when achieving the identified performance target. Finally, we also consider additional performance or safety related metrics for each of the transmission types; namely degrees of freedom for the mechanical transmission, and voltage levels for the electrical transmission.

### Mechanical Performance Targets for Surgical Manipulations

This analysis is meant to provide suitable models and parameters to aid in the design of transmissions for minimally invasive surgical robots. Therefore, it is useful to establish a mechanical performance target that, if the transmission met this target, there would be a reasonable assumption that a surgery could be carried out.

Mechanical power requirements of surgical manipulation tasks are not stated directly in the literature, though we can use independent reported task measurements to estimate an upper bound. The BlueDRAGON system has been used to measure surgeon motions and interaction forces during minimally invasive tasks (Markvicka, 2014), and reports mean and SD handle velocities (about the trocar) of 0.047 rad/s ± 0.056 rad/s while grasping during a bowel handling task (Brown et al., 2004). The forces measured, however, were at the tool handle, so are not representative of the tool/tissue interaction forces. Wagner et al. reports a histogram of forces for a minimally invasive gall bladder blunt dissection task, where all forces with a duration longer than 100 ms were below 10 N (Wagner et al., 2007).

If we assume a distance of 0.15 m from port to tool tip in (Brown et al., 2004) (half the length of a standard MIS tool shaft), and calculate the velocity that accounts for 95% of all grasp motions, this gives

(38) $$\begin{array}{ll} v\;\;\;\;\;\;\; & =\left(0.047+2\times 0.056\right)\times 0.15\\ & =0.024m/s \end{array}$$

Combining this upper bound velocity with the upper bound force limit gives a conservative upper bound mechanical power target for continuous manipulation of

(39) $$\begin{array}{ll} P_{target}\;\;\;\; & =Fv\\ & =10\times 0.024\\ & =0.24W \end{array}$$

We will use this coarse power target estimate as a baseline to compare the achievable power limits of the mechanical and electrical transmissions. We emphasize that this estimate is a continuous power upper bound; peak power demands may exceed these values.

### Heat Limits in Tissue

**Figure 4:**
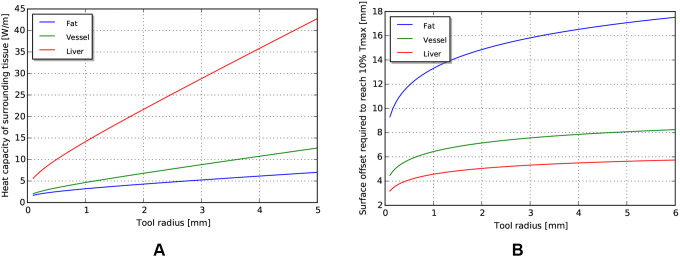
**(A)** Heat capacity of tissue for different radii of tools. **(B)** Corresponding distances away from tool at which temperature returns to 10% of maximum.

The fundamental limit to power delivery into the body relates to efficiency of the corresponding transmission, and the ability of the body to dissipate the excess heat. Using representative tissue thermal properties (Table 4), and the maximum allowed body temperature of 43 degrees C in steady state (EN 60601-1, Clause 11.1), we can derive heat limits for different radii of cylindrical tool transmissions (Figure 4A). This model assumes sufficient tissue surrounding the cylinder to dissipate the heat (Figure 4B) - if this amount of tissue is not available, more stringent limits should be used. We observe that the amount of heat that tissue can dissipate around a cylinder depends significantly on the type of tissue, and varies somewhat linearly with radius.

**Table 4:** Tissue thermal properties from (Hasgall et al., 2015).

Tissue	Perfusion	Blood mass flow ($\omega \cdot \rho_{b}$)	Thermal conductivity (k)
Fat	Low	0.521	0.211
Blood vessel wall	Medium	2.93	0.462
Liver	High	16.2	0.519

### Mechanical Tendon/sheath Transmission for MIS Surgical Robotics

#### Efficiency

Because the main efficiency expression (9) relates many effects, we explore the relative magnitude of these effects by choosing a plausible operating point (Table 5) for MIS surgical robotics, then vary individual parameters around that operating point. We consider a transmission that might be used for a colonoscopy procedure - a relatively long access path through the bowel of 0.5 m, with a continuous path radius of 0.1 m. We assume use of stainless steel wire rope as the tendon material, with a low coefficient of friction against the sheath (using the stainless steel/Teflon coefficient of friction of 0.04). We assume a tendon pull of distance 0.01 m, and a similar tendon radius (0.22 mm) as cables used on current surgical robotic tools (Friedman, 2011), as well as similar pretension (9% of working limit, approx 3.2 N).

**Table 5:** Default parameters for tendon/sheath efficiency evaluation.

Parameter	Symbol	Value
Path radius	R	0.1 m
Path length	L	0.5 m
Friction coefficient	μ	0.04
Tendon Young’s modulus	E	$97.0\times 10^{9}\;N/m^{2}$
Pull distance	D	0.01 m
Tendon radius	$r_{tendon}$	0.22 mm
Pretension	$T_{0}$	3.2 N

**Figure 5:**
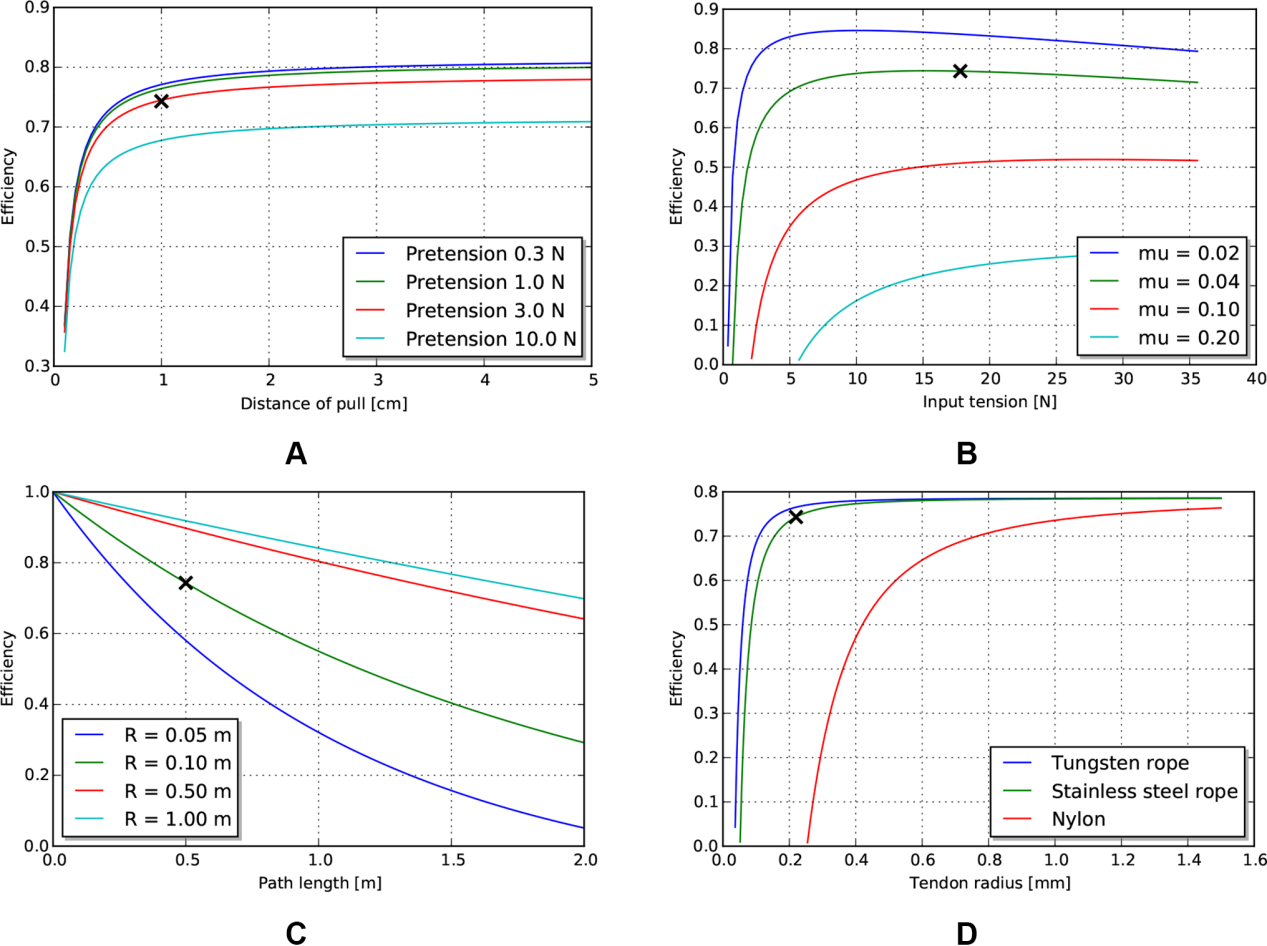
Effects of various tendon/sheath design parameters on efficiency, for default parameters given in Table 5. X’s represent parameters used in other graphs. **(A)** Effects of pretension and pull distance. **(B)** Effects of input tension and friction coefficient. **(C)** Effects of path length and radius. **(D)** Effects of tendon material and size.

The model reveals several interesting trends relating design parameters to efficiency, beyond those intuitively expected. First, increasing pretension decreases efficiency (Figure 5A), which is a tradeoff with other design effects such as tolerancing and backlash. Low force and low distance motions suffer in efficiency (and increasing burden on control), as losses due to cable stretch and friction dominate work delivered (Figure 5B). Path length and radius both serve to reduce efficiency, but as a ratio (Figure 5C). Finally, stiffness of the tendon from material property and cross sectional area serves to increase efficiency (Figure 5D).

#### Degrees of freedom

**Figure 6:**
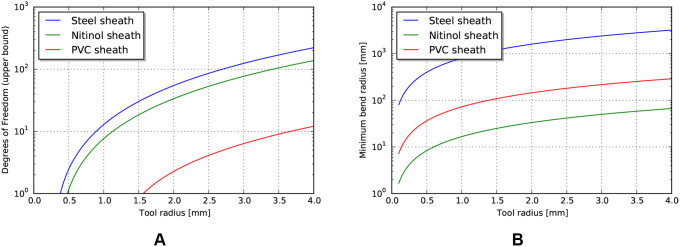
**(A)** Upper bound of degrees of freedom for tendon/sheath system described above. **(B)** Corresponding minimum achievable bend radius for sheath system.

An estimate of maximum degrees of freedom, using the design parameters listed in Table 5 is shown in Figure 6. This is an estimate of the achievable degrees of freedom using the *same* tendon radius as the operating point described above, so a similar per-degree of freedom performance. Similarly, if we make the assumption that the sheath material is continuous, then the achievable minimum bend radius can be calculated using estimates of strain at yield (Figure 6B).

Examining these results, the achievable degrees of freedom increase exponentially for a small increase in tool radius. However, the predicted minimum bend radius for the listed materials also increases exponentially. This model result highlights a common design principle: the tradeoff between the performance benefits achieved by stiff materials and the corresponding achievable minimum bend radius. Strategies to mitigate this usually involve trading off some amount of performance, or increasing the overall tool radius (to add material cross sectional area to achieve axial stiffness) but incorporating small radius articulating regions to achieve lower bend radius. Note that the large achievable strain and relatively high stiffness make nitinol an ideal candidate for monolithic sheaths.

#### Power and force limits

**Figure 7:**
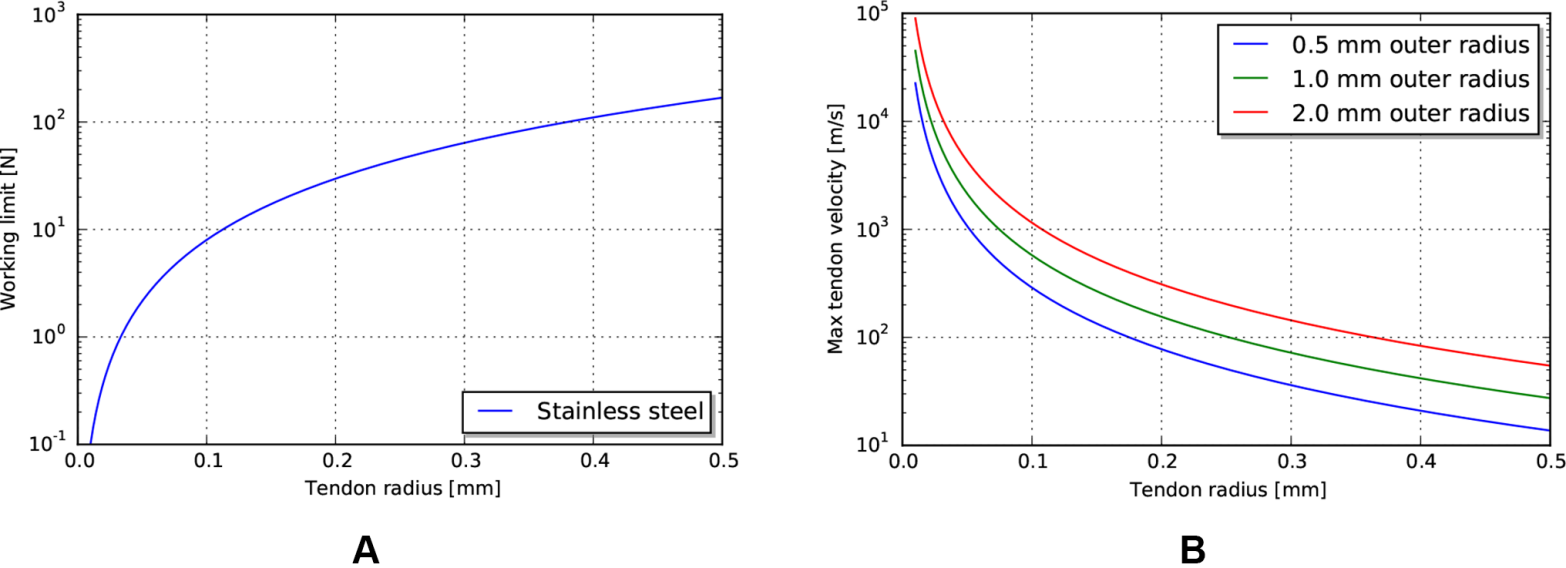
**(A)** Working limit for stainless steel wire rope by diameter. **(B)** Maximum tendon velocity at the working limit of force that would cause heat damage to the body.

The required velocity to achieve the heat-limited power throughput increases exponentially as the tendon radius decreases (Figure 7B). For the operating geometry and materials listed above, the velocities needed to reach heat damage to tissue are significantly higher than our target tool tip velocity of 24 mm/s. However, the working limit of tendons, at the sizes that are currently used in tools, is close to the 10 N force target (Figure 7A). This indicates that mechanical transmissions, for this size scale and application, are largely force limited, not power limited.

### Electrical Transmission for MIS Surgical Robotics

Using the electrical transmission models derived previously, we examine the performance of an electrical transmission under the same access constraints and heat limits as used for the mechanical tendon/sheath investigation. We also investigate current and voltage limits, as these can relate to other design considerations such as actuator compatibility and safety.

#### Current and voltage limits

**Figure 8:**
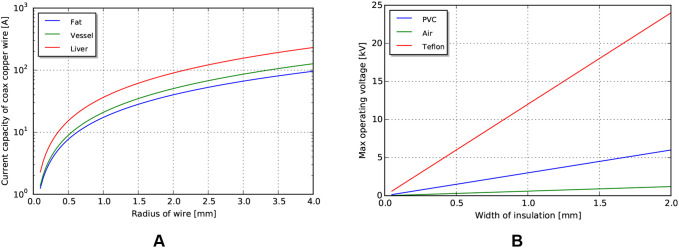
**(A)** Current limits for copper wire passing through tissue. **(B)** Voltage limits by coaxial insulation width, assuming a safety factor of 5.

Using similar tissue properties as above, we can find current limits for different radii of the coaxial wire as limited by the heat properties of tissue (Figure 8A). Current scales somewhat linearly with outer radius for the size scales relevant to MIS surgery.

Voltage limits can be determined using cross sectional area, insulator dielectric properties, and an assumed safety factor. For this analysis, we use a safety factor of 5 to match that used in the mechanical analysis. Achievable voltages for PVC (dielectric constant 15kV/mm) and PTFE (dielectric constant 60kV/mm), biocompatible insulator materials with a range of formulations for flexibility and toughness, are shown in Figure 8B, with an air gap (dielectric constant 3.0kV/mm) insulator for reference. Note that significant voltages can be achieved even with a small insulation width.

### Power and efficiency limits

**Figure 9:**
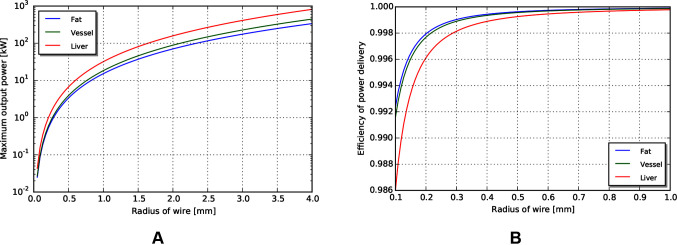
**(A)** Maximum power output for coax wires by radius. **(B)** Efficiency of wire transmission for wires of given radius.

With the same path length and tissue properties as used in the above mechanical transmission analysis, we can estimate the maximum power output for a coaxial cable with similar access constraints. Assuming a copper cable (with resistivity of $1.68\times 10^{-8}\;\Omega m$) with PVC insulation, the maximum power achievable for a 0.5 m wire length is shown in Figure 9A. Note that the maximum power capacity for this coaxial wire far exceeds the mechanical power requirements estimated earlier.

Similar to above, the efficiency in different tissues across outer radius is shown in Figure 9B. We observe that the efficiency of a wire transmission can exceed 99%, even for wires with l = 1 m and r < 0.2 mm, and subject to safety constraints within the body.

## Discussion

The work presented identifies the efficiency limits of transmissions used for small access diameter surgical robotics. We present efficiency and power limit models for tendon/sheath mechanical transmissions and electrical wire transmissions derived from first principles and from existing models in the literature. The models incorporate access constraints as well as safety limits required when implementing surgical systems in a medical device design context. We then used the models to investigate achievable power and efficiency limits using representative values for minimally invasive robotic surgery.

The results of the analysis validated the initial hypothesis - for longer path lengths, higher path curvature, and higher pretension, the mechanical transmission decreased in efficiency. A key observation is that the decrease was significant in the size scales and parameters relevant for MIS surgery. Mechanical transmissions achieved 85% efficiency for shorter, straighter paths, to below 25% for longer, curved paths with higher pretension.

For similar path geometries and access constraints, electrical coaxial wire (coax to provide a current return path and an isolation barrier) achieved high efficiencies - over 99% efficient power transfer, even with small diameter access. Again, the key observation is that this efficiency result holds for the sizes relevant to MIS surgical robotics. An important qualification, however, is that this efficiency does not take into account the efficiency of the actuator, which is likely to be lower than the corresponding efficiency of the mechanism required to convert tendon motion into tool motion. We discuss this further in the next section.

We also investigated the ability of the two transmissions to deliver other performance criteria required for surgical manipulations beyond efficiency; specifically, force, velocity and power. We observed that, for current materials used in tendon/sheath construction, the tendon working limit is near the force limit required for surgical manipulations. The velocities of the cables at those working limits, however, are much smaller than those imposed by the heat limit, implying the ability of a tendon/sheath system to deliver significantly more absolute power if tendon velocities are increased. Thus, tendon/sheath transmissions will struggle to deliver the same performance for longer access paths or smaller cross-sectional area if tendons are used in the same manner (at forces and velocities similar to those needed for surgery).

The electrical transmission, from an absolute power delivery standpoint, has the ability to deliver significantly more power than required for surgical manipulations. For example, a 1 mm radius coaxial wire as described can deliver over 10 kW of power in a safe manner, which is many orders of magnitude above the target 0.25 W mechanical power needed for surgical manipulations. Again, the limiting factor in this case would be the actuator, not the transmission, and 10 kW of continuous power could not be dissipated by the body. However, this highlights the potential opportunity for smaller MIS robots, given sufficient actuation technology.

### Candidate Actuators for Locating Inside the Body

The results presented show that the efficiency of a wire to transmit power is high even under the access constraints posed by MIS surgery; however, creating a full minimally invasive surgical robot based on an electrical transmission requires locating the actuators on the inside of the body. While a complete analysis of optimal actuator technology for surgical robotics is out of scope for this paper, it is worthwhile to mention candidate actuation technologies with sufficient work-density characteristics that could enable practical implementations of existing surgical tools.

Overviews of actuator technology show several smart material actuation technology that are significantly more work dense than traditional moving coil actuators (Huber et al., 1997). These include piezoelectric actuators and shape memory alloy. Piezoelectric technology is both significantly more work dense than traditional motors (up to $10^{9}$$W/m^{3}$ as compared to $2\times 10^{6}$ $W/m^{3}$) and more efficient (above 99% as compared to 50–80%). However, the drawback is that the strain achieved per stroke is small, so some additional transmission would be required to convert the output into forces and displacements useful for the task.

Similarly, shape memory alloy has a higher work density than moving coil transducers (up to $10^{8}$ $W/m^{3}$), but has some drawbacks. Primarily, shape memory alloys rely on a thermal effect to generate actuation, and so are poor in terms of efficiency (1–2%). Ongoing research, however, is examining approaches for increasing the efficiency and thermal properties of shape memory alloy (Thrasher et al., 1994; Pathak, 2010; Nespoli et al., 2010; Salerno et al., 2014; Khan et al., 2016), making it a better candidate for an internal actuator.

MIS robotic manipulators incorporating both miniature moving coil actuators and SMA actuators have been developed. Mineta et al. and Takayama et al. (Takayama et al., 1997; Mineta et al., 2001) developed catheter-like manipulators incorporating SMA actuators. In their work they showed it is possible to practically incorporate SMA actuators in MIS positioners that could for example guide a monopolar electrosurgery tool for dissection and cauterisation operations. Hideki and Salerno (Okamura et al., 2009; Salerno et al., 2014) explored the possibility of employing SMA actuators in robotic grippers with encouraging results. Lee et al. and Yeung et al. (Yeung and Gourlay, 2012; Lee et al., 2014) designed small robotic tools with moving coil actuators integrated in the tool and robot body and could apply up to 10 N of grasping force. This strategy could be better suited for tools such as grippers and needle drivers, and even staplers with an appropriate reduction ratio.

### Model Limitations

Our investigation focused on the power and efficiency limits of power transmission into the body. These are not the only source of limiting factors to consider, however, when designing a surgical robot. One of the main omissions of the previous analysis is establishing a mechanical ground against which to apply force. There are a number of solutions that exists whose application depends on the specific surgical manipulation. For example, multiple robots entering the body from different ports can increase stiffness of the base (Mahoney et al., 2017). Additionally, forces internal to the robot do not require an external mechanical ground, so multiple armed systems can be effective. Finally, the robot can use alternative anchoring strategies, such as cuffs or balloons, to establish mechanical ground distally.

The efficiency limits developed for tendon/sheath transmissions considered a number of material property and geometry effects. Two similar effects not incorporated include the stiffness of the sheath material and the effects of the surrounding tissue. If these stiffnesses were low compared to the tendon stiffness, these would further reduce the observed efficiency of the system. Further, we didn’t consider effects of any secondary transmissions, such as a pulley at the distal end to enable jaw rotation.

Similarly, we also did not consider all electrical transmission effects. We assumed a DC current in our analysis, but AC current may be more practical, depending on the actuator technology. If AC power delivery was warranted, then skin depth effects (and known mitigations such as Litz wire) should also be considered. Further, we did not account for data transmission effects - if the same conductor was used to transmit control commands as well as power, conductor effects might limit data transmission bandwidth which could potentially limit the available degrees of freedom.

### Future Directions

The results for the mechanical tendon/sheath transmission showed that current systems work close to the force limits of the materials, but far away from the heat limits of the body. This implies that more power can be transmitted into the body for the same access geometry, if the transmission operated at higher velocities. This comes with its own set of challenges - if a tendon is operating with a direct link to an output degree of freedom, changes in desired output direction would require a change in direction of a high velocity tendon. This would require low backlash and low tendon inertia; properties that are difficult to achieve with today’s tendon materials. Further, that high tendon velocity would need to be converted into a lower velocity output motion, requiring an additional transmission (in the gearing sense) at the output.

This leads to the related consideration of busing - using the same power delivery line for multiple degrees of freedom. Or, put another way, allowing all of the power delivery potential of the transmission cross sectional area to pass through a single degree of freedom. This is straightforward to imagine in the case of electrical transmission to multiple switched actuators, but more difficult to envisage in the mechanical context. One mechanical busing scheme to consider is a hydraulic transmission, with a series of controlled valves to gate power to the corresponding degree of freedom. Identifying the control and valve technology remains a challenge, but hydraulics have the advantage of graceful failure modes on puncture - assuming biocompatible hydraulic fluids and a sufficiently stiff delivery tube, high pressures will quickly dissipate as the working fluid is incompressible. Mechanical busing could potentially change how power is delivered mechanically, which is not force limited, and could allow transmission around an operating point of peak efficiency.

The electrical efficiency models presented in this work motivate the opportunity for continued investigation into sufficiently efficient and work-dense electrical actuators for MIS surgical robotics. Correct use of these actuators can enable equal or better mechanical performance as mechanical cable drives, with little of the external mechanical infrastructure and size required. This points to a vision for the future of surgical robotics, where all of the MIS benefits can be delivered with a small robotic system.

Finally, we observe that for both mechanical and electrical transmissions, efficiency estimates indicate that current systems are far from the allowable continuous heat limits that can be safely accounted for by the body. This highlights the opportunity to further decrease access size, and increase the range of procedures that the benefits of surgical robotics can be applied to.

## Author Contributions

CW derived models, carried out the analysis, and was the primary author of the text. EE carried out additional analysis, verification of results, and contributed to the text. Both were involved in developing the underlying ideas and direction of the work, revising the manuscript, and approving the submitted version.

## Conflict of Interest Statement

Both authors were employed by the company Cambridge Consultants, Ltd during the writing of the paper.

## Appendix

### Heat propagation through a cylinder

Here we present the derivation of an analytic model of the local heat propagation from a cylinder (such as a wire) into surrounding tissue in steady state using the bioheat equation (Incropera et al., 2011). The derivation of the presented here initially follows the one presented by Yue et al (Yue et al., 2004), a solution of the bioheat equation in steady state in cylindrical coordinates. We solve for different boundary conditions, however, allowing the analysis of the heat flux through the heat-generating wire. Several main results are presented here: (1) the analytic solution to the bioheat equation given boundary conditions of two known temperatures at fixed radii, (2) a simplification of this result with the outer radius set at infinity, and (3) the corresponding heat flux through the wire given a temperature constraint to nearby tissue. Please see Table 1 for nomenclature used.

More formally, given a cylindrical pipe of tissue with outer radius $r_{2}$ and inner radius $r_{1}$, and with known temperatures at the radii $T_{2}$ and $T_{1}$ respectively, determine the corresponding heat flow into the cylindrical pipe through the inner radius, at steady state.

We start with the bioheat equation in cylindrical coordinates for one dimension, in steady state, given by:

(40) $$\frac{1}{r}\frac{d}{dr}\left(r\frac{dT}{dr}\right)+\frac{\omega \rho_{b}c_{b}}{k}\left(T_{a}-T\right)+\frac{{\dot{q}}_{m}}{k}=0$$

From this equation, temperature T at a particular radius r is related through the main effects of diffusion, perfusion from capillary action, and metabolic heat (${\dot{q}}_{m}$). Other parameters include ω the perfusion rate [$m^{3}/(s\cdot m^{3})$] of blood through capillaries in a particular tissue, $\rho_{b}$ the density ($kg/m^{3}$) of blood, $c_{b}$ the specific heat [J/(kg⋅K)] of blood, k the tissue thermal conductivity [W/(m⋅K)], and $T_{a}$ arterial blood temperature.

To have a safe limit that applies even if the tissue is deep within the body, we are deriving a solution such that all of the heat put into the system through $r_{1}$ is removed through perfusion effects. This has the advantage of avoiding a convective boundary condition, which are difficult to parameterise for internal tissue interactions. Also, we assume steady state to analyze the worst case limit of continual heat input into the body. We set the metabolic term ${\dot{q}}_{m}$ to 0 for this analysis, as its effect will be negligible.

To solve (40), first use a series of substitutions to rewrite the equation into a differential equation form with a known solution. If we let

(41) $$\begin{array}{c} B=\sqrt{\frac{\omega \rho_{b}c_{b}}{k}}\\ A=B^{2}T_{a}\\ \Phi (r)=A-B^{2}T(r) \end{array}$$

and substitute into the bioheat equation (40), we are left with a differential equation of the form

(42) $$\frac{d^{2}\Phi }{dr^{2}}+\frac{1}{r}\frac{d\Phi }{dr}-B\Phi =0$$

By inspection, this is a zero-order modified Bessel differential equation with known solution given by

(43) $$\Phi \left(r\right)=C_{1}I_{0}\left(Br\right)+C_{2}K_{0}\left(Br\right)$$

where $I_{0}$ is a zeroth order modified Bessel function of the first kind and $K_{0}$ is a zeroth order modified Bessel function of the second kind. To solve for the constants $C_{1}$ and $C_{2}$, we use the boundary conditions of known temperature at specified radii

(44) $$T(r_{1})=T_{1}$$

(45) $$T(r_{2})=T_{2}$$

along with the above substitutions (41). Solving the two resulting equations gives

(46) $$C_{1}=\frac{B^{2}\left(-T_{1}K_{0}\left(Br_{2}\right)+T_{2}K_{0}\left(Br_{1}\right)-T_{a}K_{0}\left(Br_{1}\right)+T_{a}K_{0}\left(Br_{2}\right)\right)}{I_{0}\left(Br_{1}\right)K_{0}\left(Br_{2}\right)-I_{0}\left(Br_{2}\right)K_{0}\left(Br_{1}\right)}$$

(47) $$C_{2}=\frac{B^{2}\left(\left(T_{1}-T_{a}\right)I_{0}\left(Br_{2}\right)-\left(T_{2}-T_{a}\right)I_{0}\left(Br_{1}\right)\right)}{I_{0}\left(Br_{1}\right)K_{0}\left(Br_{2}\right)-I_{0}\left(Br_{2}\right)K_{0}\left(Br_{1}\right)}$$

Substituting this result into (43) gives the analytic solution for the bioheat equation in steady state, for fixed temperature boundary conditions:

(48) $$\begin{array}{c} T\left(r\right)=\frac{T_{a}\left(I_{0}\left(Br_{1}\right)K_{0}\left(Br_{2}\right)-I_{0}\left(Br_{2}\right)K_{0}\left(Br_{1}\right)\right)}{I_{0}\left(Br_{1}\right)K_{0}\left(Br_{2}\right)-I_{0}\left(Br_{2}\right)K_{0}\left(Br_{1}\right)}\\ -\frac{\left(T_{1}-T_{a}\right)I_{0}\left(Br_{2}\right)-\left(T_{2}-T_{a}\right)I_{0}\left(Br_{1}\right)}{I_{0}\left(Br_{1}\right)K_{0}\left(Br_{2}\right)-I_{0}\left(Br_{2}\right)K_{0}\left(Br_{1}\right)}K_{0}\left(Br\right)\\ +\frac{T_{1}K_{0}\left(Br_{2}\right)-T_{2}K_{0}\left(Br_{1}\right)+T_{a}K_{0}\left(Br_{1}\right)-T_{a}K_{0}\left(Br_{2}\right)}{I_{0}\left(Br_{1}\right)K_{0}\left(Br_{2}\right)-I_{0}\left(Br_{2}\right)K_{0}\left(Br_{1}\right)}I_{0}\left(Br\right) \end{array}$$

However, using this solution directly can lead to unrealistic results when trying to derive heat flux limits at $r_{1}$, as nothing in the solution limits the heat flux at $r_{2}$. To overcome this limitation, solve (48) in the limit as $r_{2}$ goes to infinity. At this limit, we can safely assume there is no external heat sink effect biasing the flux calculation at $r_{1}$, and all heat removal contributions are from perfusion and diffusion. Given that we will use the maximum safe steady state temperature in the body at $r_{1}$, the heat power limit estimate will hold given that the temperature falls off within reasonable distances.

Knowing that $lim_{x\to \infty }I_{0}(x)=\infty$ and $lim_{x\to \infty }K_{0}(x)=0$ lets us derive the following expression for $lim_{r_{2}\to \infty }T_{full}(r)$ by inspection, which we term $T_{inf}\left(r\right)$:

(49) $$T_{inf}\left(r\right)=T_{a}+\frac{T_{1}-T_{a}}{K_{0}\left(Br_{1}\right)}K_{0}\left(Br\right)$$

$T_{inf}(r)$ is the analytic solution of the bioheat equation given no outer radial constraint on temperature. Note that for this limit, $T_{2}$ is not a parameter - it naturally falls out as body temperature ($T_{a}$).

Using $T_{inf}(r)$, we can now use Fourier’s law of heat conduction to relate the rate of temperature change (dT/dr) to the power flux ($\dot{Q}$) due to heat through $r_{1}$. To be clear on units, we are using $\dot{Q}$ to denote heat power (in units of Watts) and $\dot{q}$ to denote heat flux in units of Watts per square meter, where $\dot{q}=\dot{Q}/a$ where a is area.

The one dimensional version of Fourier’s law, in cylindrical coordinates, is:

(50) $$\dot{q}=-k\frac{dT}{dr}$$

where k is the thermal conductivity. Substituting in for heat flux gives:

(51) $$\frac{\dot{Q}}{a}=k\frac{dT}{dr}$$

Because we are solving for heat flux through a cylinder, use a=2πrl, where l is the length of the cylinder.

(52) $$\frac{\dot{Q}}{2\pi rl}=k\frac{dT}{dr}$$

Then, rearrange to find $\dot{Q}/l$, which is the heat power per unit length of the cylinder, which we will define as H(r):

(53) $$H\left(r\right)=\frac{\dot{Q}}{l}=2\pi rk\frac{dT}{dr}$$

This is the heat power that is transmitted through a cylinder, per unit length, for a given temperature differential and thermal conductivity *k*. Carrying out the derivative $\frac{dT_{inf}\left(r\right)}{dr}$ and substituting into (53) gives

(54) $$H_{max}=2\pi kBr_{1}\frac{K_{1}(Br_{1})}{K_{0}(Br_{1})}(T_{1}-T_{a})$$

where

(55) $$B=\sqrt{\frac{\omega \rho_{b}c_{b}}{k}}$$

which is an analytic result to estimate the maximum heat power (in W/m) that specific tissues in body can can safely dissipate for a given cylindrical geometry.

### Tendon/sheath transmission force balance

Here we derive a simple tendon/sheath model that relates input and output tensions as parameterised by a radial path geometry, and a friction coefficient relating tendon tension with friction forces. This is the same model as presented by Palli et al. (Palli and Melchiorri, 2006; Palli et al., 2009), but we re-derive the solution to the model to be explicit about the contribution of pretension (not in the original derivation) so that it can be correctly incorporated into an expression for efficiency.

As stated in (Palli and Melchiorri, 2006), we model the force balance of a small section of tendon (Figure 2):

(56) $$\Delta T=-F_{f}=-\mu Nsign\left(\dot{\epsilon }\right)$$

where ΔT is the change in tension, $F_{f}$ is the force due to friction, μ is the friction coefficient, and N is the normal force. $sign\left(\dot{\epsilon }\right)$ ensures that friction force is acting opposite the direction of motion of the tendon. We will neglect this direction term for the remaining analysis, as we are focusing on deriving an expression for efficiency limits which can be derived sufficiently from a single-direction analysis.

The normal force N is given by:

(57) $$N=T\Delta \gamma =T\frac{\Delta x}{R}$$

where Δγ is the subtended angle, Δx is the length, and R is the radius of curvature of the tendon element. Then, for an infinitesimal section of tendon:

(58) $$dT=-\mu T\frac{dx}{R}$$

Solving for T(x) gives

(59) $$T(x)=T_{in}e^{-\frac{\mu x}{R}}$$

where $T_{in}$ is the input tension at x=0.

Similarly, to solve for the cable stretch δ as a function of distance, we start with the stretch along the tendon section:

(60) $$\Delta \delta =T(x)\frac{\Delta x}{EA}$$

where E is the elasticity of the tendon material, and A is the tendon cross sectional area. For the infinitesimal tendon section, the differential equation becomes:

(61) $$\frac{d\delta }{dx}=\frac{1}{EA}T(x)$$

whose solution is:

(62) $$\delta (x)=\frac{T_{in}R}{EA\mu }\left(1-e^{-\frac{\mu x}{R}}\right)$$

Thus, our model to relate tensions and tendon stretch, for a tendon of length L, path radius R, and friction coefficient μ is:

(63) $$\begin{array}{c} T_{out}=T_{in}e^{-\frac{\mu L}{R}}\\ \delta =\frac{T_{in}R}{EA\mu }\left(1-e^{-\frac{\mu L}{R}}\right) \end{array}$$

Again, this model accounts for tension and stretch for single direction motions, but does not account for hysteresis effects when tendon motion reverses direction.

Now, we introduce an explicit pretension term $T_{0}$ as a component of both input and output tension, where $T_{w}$ is the remaining tension that is doing work:

(64) $$\begin{array}{c} T_{in}=T_{in,w}+T_{0}\\ T_{out}=T_{out,w}+T_{0} \end{array}$$

Solving for $T_{out,w}$ is then

(65) $$\begin{array}{ll} T_{out,w} & =T_{out}-T_{0}\\ & =T_{in}e^{-\frac{\mu L}{R}}-T_{0}\\ & =\left(T_{in,w}+T_{0}\right)e^{-\frac{\mu L}{R}}-T_{0}\\ & =T_{in,w}e^{-\frac{\mu L}{R}}-T_{0}\left(1-e^{-\frac{\mu L}{R}}\right) \end{array}$$

We also carry out the same separation for the tendon stretch δ into a prestretch term $\delta_{0}$ and the stretch due to the input work tension $\delta_{w}$:

(66) $$\begin{array}{ll} \delta_{w} & =\delta -\delta_{0}\\ & =\frac{\left(T_{in,w}+T_{0}\right)R}{EA\mu }\left(1-e^{-\frac{\mu L}{R}}\right)-\frac{T_{0}R}{EA\mu }\left(1-e^{-\frac{\mu L}{R}}\right)\\ & =\frac{T_{in,w}R}{EA\mu }\left(1-e^{-\frac{\mu L}{R}}\right) \end{array}$$
